# Model Reduction Through Progressive Latent Space Pruning in Deep Active Inference

**DOI:** 10.3389/fnbot.2022.795846

**Published:** 2022-03-11

**Authors:** Samuel T. Wauthier, Cedric De Boom, Ozan Çatal, Tim Verbelen, Bart Dhoedt

**Affiliations:** IDLab, Department of Information Technology, Ghent University—imec, Ghent, Belgium

**Keywords:** active inference, free energy, deep learning, model reduction, generative modeling

## Abstract

Although still not fully understood, sleep is known to play an important role in learning and in pruning synaptic connections. From the active inference perspective, this can be cast as learning parameters of a generative model and Bayesian model reduction, respectively. In this article, we show how to reduce dimensionality of the latent space of such a generative model, and hence model complexity, in deep active inference during training through a similar process. While deep active inference uses deep neural networks for state space construction, an issue remains in that the dimensionality of the latent space must be specified beforehand. We investigate two methods that are able to prune the latent space of deep active inference models. The first approach functions similar to sleep and performs model reduction *post hoc*. The second approach is a novel method which is more similar to reflection, operates during training and displays “aha” moments when the model is able to reduce latent space dimensionality. We show for two well-known simulated environments that model performance is retained in the first approach and only diminishes slightly in the second approach. We also show that reconstructions from a real world example are indistinguishable before and after reduction. We conclude that the most important difference constitutes a trade-off between training time and model performance in terms of accuracy and the ability to generalize, *via* minimization of model complexity.

## 1. Introduction

While the role of sleep in animals still contains a lot of mystery (Mignot, 2008; Joiner, 2016), it has been linked to many phenomena, such as restorative processes in the brain (Hobson, 2005) and memory processing (Born and Wilhelm, 2012; Potkin and Bunney, 2012; Stickgold and Walker, 2013). In particular, sleep and learning appear to be deeply intertwined (Korman et al., 2003; Tononi and Cirelli, 2006; Holz et al., 2012). Recent work has indicated that the removal of redundant neural connections during sleep (Li et al., 2017) can be compared to minimization of complexity through elimination of redundant parameters during Bayesian model reduction (BMR) in Bayesian approaches to brain function (Hobson and Friston, 2012; Friston et al., 2017b, 2019). Removal of redundant connections while strengthening others should promote learning (Li et al., 2017).

Artificial agents used for learning specific tasks are often based on the formalism of Markov decision processes (MDPs) (Watkins, 1989; Mnih et al., 2015; Hafner et al., 2019). In this formalism, the complexity of the environment determines the complexity of the latent space (a.k.a. state space), as the number of dimensions grows rapidly with the number of possible states the agent can find itself in. A large state space increases computational costs and can lead to overfitting (Sỳkora, 2008). It is possible to reduce the state space through various methods, such as state clustering or segmentation [e.g., Q-learning with adaptive state segmentation (Murao and Kitamura, 1997)], state vector transformation [e.g., basis iteration for reward based dimensionality reduction (Sprague, 2007)] and state space reconstruction [e.g., action respecting embedding (Bowling et al., 2005; Sỳkora, 2008)]. State space reduction can improve generalization, as well as reduce computational complexity and learning time.

In cases where the agent cannot fully observe the underlying state of the world, the formalism is generalized into a partially observable Markov decision process (POMDP) and decisions cannot be made based on the current state, but must be made based on the current belief about the state. For example, when the agent's observations consist of images, there are a number of variables which are not exactly known, such as the agent's own velocity, the velocity of objects in the images, the existence of objects outside the field of view of the camera, etc. The agent can, however, infer some of these variables based on the information in the images and, as a result, form beliefs about these variables. Which variables are relevant depends strongly on the task at hand. Therefore, it is inefficient to always attempt to track every possible variable. A possible solution exists in the form of feature learning to extract a smaller size feature space, which can then be used as state space. While deep neural networks have been shown to be rather good at encoding high-dimensional (observation) spaces into low-dimensional (feature) spaces (Hinton and Salakhutdinov, 2006), the issue remains that the size of the low-dimensional space must be specified. This space should be small enough to promote generalization and reduce complexity, yet large enough to maintain model accuracy.

In this article, we focus on pruning the latent space in deep active inference. Active inference is a theory of behavior and learning that has been gaining attention in the last decade (Friston et al., 2016; Kirchhoff et al., 2018; Millidge et al., 2020; Sajid et al., 2021). The theory adopts the Bayesian brain hypothesis and, as such, frames sleep as Bayesian model reduction. Recently, methods have been developed through which the latent space is learned by deep neural networks. These methods are known as deep active inference (Ueltzhöffer, 2018; Çatal et al., 2019) and have been shown to be able to solve multiple environments (Çatal et al., 2020), i.e., the mountain car problem, the OpenAI Gym car racing environment (Brockman et al., 2016) and a robot navigation environment.

We present two methods for latent space pruning in the deep active inference framework. First, we discuss the off-line algorithm from our earlier work (Wauthier et al., 2020), which acts on a model which has previously been trained. Second, we present an on-line algorithm that prunes dimensions on-the-fly during training on sequences of observations and actions. We explore some of the properties of the latter and compare both methods in terms of performance and show that both methods are able to effectively prune dimensions in the latent space. Furthermore, we show that the off-line method requires a longer training time, but maintains performance, while the on-line method requires a shorter training time, but leads to a slight drop in performance.

This work is structured as follows. In Section 2, we provide an overview of related work. In Section 3, we briefly summarize (deep) active inference and describe our methods for pruning. We proceed with a description of the environments used in the experiments. In Section 4, we explain the experiments and display the results, which we discuss in Section 5. In Section 6, we conclude this work and examine future prospects.

## 2. Related Work

The earliest use of the term “active inference”, as it is used now, in relation to the free energy principle (Friston et al., 2006) dates back to Friston et al. (2009). Since then, it has been expanded upon in a number of ways and applied in a multitude of scenarios. Beside continued work on behavior and learning (Friston et al., 2015, 2016), active inference has been described as a process theory (Friston et al., 2017a) and as a hierarchical model (Friston et al., 2018). Furthermore, active inference has been employed in hermeneutics (Friston and Frith, 2015), in a theory of allostasis (Barrett et al., 2016), to augment traditional reinforcement learning approaches (Tschantz et al., 2020), and in humanoid robot control (Oliver et al., 2021). In particular, our methods relate to developments in deep active inference (Ueltzhöffer, 2018; Çatal et al., 2019, 2020), where the goal is to step away from predefined state spaces by learning through artificial neural networks.

Thus far, sleep has yet to be covered extensively in the context of active inference. Hobson and Friston (2012) review the purpose of sleep in terms of free energy minimization. Similarly, the premise that the brain's generative model is actively refined during sleep is explored by Hobson et al. (2014). Friston et al. (2017b) detail the parallels between Bayesian model reduction and certain mechanisms associated with sleep or “Aha” moments. Specifically, it refers to the removal of redundant connections to minimize complexity. Further, sleep has been tied to concept learning, where Bayesian model reduction merges different states into one and reduces complexity (Smith et al., 2020).

In comparison, artificial neural network pruning has a relatively long history. As a means to improve generalization and reduce hardware and storage requirements, Le Cun et al. (1989) suggested a method for removing unimportant weights from a neural network using second derivatives. In response, Hassibi and Stork (1992) proposed a method using the inverse Hessian matrix from training data and structural information of the neural network. In recent years, with the popularity of deep neural networks, the discussion has been reopened. Various techniques have been developed to compress large models and reduce the number of parameters. Gong et al. (2014) examine information theoretical vector quantization methods. Han et al. (2016) introduced a 3-step method involving pruning, trained quantization and Huffman coding. Other notable contributions include “soft weight-sharing” (Ullrich et al., 2017), variational dropout (Molchanov et al., 2017), *L*_0_-norm-based methods (Louizos et al., 2018; Li and Ji, 2020), and Dirichlet pruning (Adamczewski and Park, 2021).

Many dimensionality reduction techniques exist for the purpose of transforming a high-dimensional space into a low-dimensional space. Traditional dimensionality reduction techniques include singular value decomposition (SVD) and the related method principal component analysis (PCA) (Pearson, 1901), linear discriminant analysis (LDA) (Cohen et al., 2003), non-negative matrix factorization (NMF) (Lawton and Sylvestre, 1971), etc. In this article, we also investigated SVD as state dimensionality reduction technique after training. The downside is that this requires retraining afterwards. A well-known neural network-based method is that of the autoencoder (Kramer, 1991), which inspired the more recent variational autoencoder (VAE) (Kingma and Welling, 2014). However, also in this case the dimensionality of the latent bottleneck is a hyperparameter that needs to be tuned by the experimenter. Model reduction methods related to Markov decision processes can be classified into three different approaches. Clustering and segmentation approaches attempt to partition the state space into a finite number of partitions, examples include Q-learning with adaptive state segmentation (QLASS) (Murao and Kitamura, 1997) and extended ϵ-reduction for hierarchical reinforcement learning (Asadi and Huber, 2004). In our case, observations can contain continuous features, which would require an infinite number of states. State vector transformation approaches project a high-dimensional space onto a low-dimensional space, such as basis iteration for reward based dimensionality reduction (Sprague, 2007), locally linear embedding (LLE) (Roweis and Saul, 2000) and dimensionality reduction by learning an invariant mapping (DrLIM) (Hadsell et al., 2006). Apart from DrLIM, these methods require recomputation of the embedding for each unknown datapoint, and rely on predetermined computable distance metrics. DrLIM, on the other hand, requires training a neural network. State space reconstruction methods build a low-dimensional representation of the data, e.g., multidimensional scaling (Borg and Groenen, 2005) and action respecting embedding (ARE) (Bowling et al., 2005). Multidimensional scaling, like PCA and SVD, generates linear embeddings. ARE is not able to embed unknown datapoints. A similar approach to the off-line sleep method performs reduction on the state space in deep Q-networks through PCA (Ge and Ouyang, 2018).

## 3. Methods

### 3.1. Active Inference

Consider an agent, natural or artificial, inhabiting an environment, e.g., a living room or a warehouse. Such an agent interacts with its environment in two ways: it can observe the state of the environment and it can perform actions within the environment. For example, a mouse can use its eyes to see where it is and use its mouth to eat cheese, or a roomba can see the shape of the room using its sensors and clean up dirt using its brushes. However, due to limitations on the sensors of the agent, such as noise and range limits, an agent can never know the exact state of the environment, e.g., the mouse cannot see what is going on outside of its field of view and the roomba cannot know the shape of the room if its view is blocked by a large piece of furniture. Therefore, it must always make decisions based on incomplete information. This concept is formalized as a partially observable Markov decision process (POMDP).

Active inference postulates that a natural agent maintains an internal model of the world (Friston et al., 2016). That is, an agent holds beliefs about the world. Mathematically, this is represented by a generative model $P\left(\tilde{o},\tilde{s},\pi \right)$ over possible observations ***o***, states ***s***, and policies π,

(1) $$\begin{array}{l} P\left(\tilde{o},\tilde{s},\pi \right)=P\left(s_{0}\right)P\left(\pi \right)\overset{T}{\underset{t=1}{\prod }}P\left(o_{t}|s_{t}\right)P\left(s_{t}|s_{t-1},\pi \right), \end{array}$$

where the tilde notation indicates a variable sequence over time, i.e., $\tilde{x}=\left(x_{1},x_{2},x_{3},\ldots ,x_{T}\right)$ and policies are sequences of actions ***a***_*t*_ = π(*t*). This generative model is continually updated to include evidence from new observations.

Active inference adheres to the free energy principle in that action selection occurs on the basis of variational free energy (Friston et al., 2016). In other words, an agent selects its actions in such a way that the actions minimize a free energy functional

(2) $$\begin{array}{l} F={\text{𝔼}}_{Q\left(s,\pi \right)}\left[\log Q\left(s,\pi \right)-\log P\left(\tilde{o},\tilde{s},\pi \right)\right] \end{array}$$

(3) $$\begin{array}{l} =\underset{complexity}{\underset{︸}{{\text{D}}_{\text{KL}}\left(Q\left(s,\pi \right)||P\left(s,\pi \right)\right)}}-\underset{accuracy}{\underset{︸}{{\text{𝔼}}_{Q\left(s,\pi \right)}\left[\log P\left(\tilde{o}|\tilde{s},\pi \right)\right]}}, \end{array}$$

where *Q* denotes the approximate posterior which emerges in variational Bayesian methods and D_KL_ denotes the Kullback-Leibler (KL) divergence. Minimizing this functional corresponds to minimizing the complexity of accurate explanations. Particularly, it constitutes a trade-off between model complexity and accuracy.

Crucially, an agent will also aim to minimize its free energy in the future, and hence select policies that it believes will yield a low free energy. This leads to the notion of expected free energy over a certain policy

(4) $$\begin{array}{l} G\left(\pi \right)=\underset{\tau }{\sum }G\left(\pi ,\tau \right) \end{array}$$

(5) $$\begin{array}{l} G\left(\pi ,\tau \right)={\text{𝔼}}_{Q\left(o_{\tau },s_{\tau }|\pi \right)}\left[\log Q\left(s_{\tau }|\pi \right)-\log P\left(o_{\tau },s_{\tau }|\pi \right)\right] \end{array}$$

(6) $$\begin{array}{c} =\underset{expected\;cost}{\underset{︸}{{\text{D}}_{\text{KL}}(Q(s_{\tau }|\pi )||P(s_{\tau }))}}+\underset{expected\;ambiguity}{\underset{︸}{{\text{𝔼}}_{Q(s_{\tau }|\pi )}[H(\log P(o_{\tau }|s_{\tau })]}}, \end{array}$$

where we have used that *Q*(***o***_τ_, ***s***_τ_|π) ≈ *P*(***o***_τ_|***s***_τ_)*Q*(***s***_τ_|π). Expected free energy again decomposes into two terms: expected cost and expected ambiguity. Expected cost is the divergence between predicted state distribution and the preferred state distribution *P*(***s***_τ_), i.e., the states the agent wants to be in. Expected ambiguity is the accuracy expected under predicted outcomes. This means that an agent will select policies that realize preferred outcomes and resolve ambiguity.

### 3.2. Deep Active Inference

Belief state spaces in active inference are typically constructed manually, and/or taken to be identical to the state space of the generative process of the modeled environment when known to the experimenter (Da Costa et al., 2020). In other words, every aspect of the state space is specified by hand. However, handcrafting a state space is typically only feasible for low-dimensional problems. The task becomes more difficult for high-dimensional problems, e.g., in the case of high-dimensional sensor input such as RGB images, and problems where the dynamics are difficult to model by hand. For that reason, recent work has focused on learning state space representations using artificial neural networks.

In this work, we adopt the model as implemented by Çatal et al. (2020). With this framework, the generative model in Equation (1) is slightly reformulated. The policy π is broken up into actions ***a***_*t*_, so that

(7) $$\begin{array}{l} P\left(\tilde{o},\tilde{s},\tilde{a}\right)=P\left(s_{0}\right)P\left(\tilde{a}\right)\overset{T}{\underset{t=1}{\prod }}P\left(o_{t}|s_{t}\right)P\left(s_{t}|s_{t-1},a_{t-1}\right). \end{array}$$

**Figure 1:**
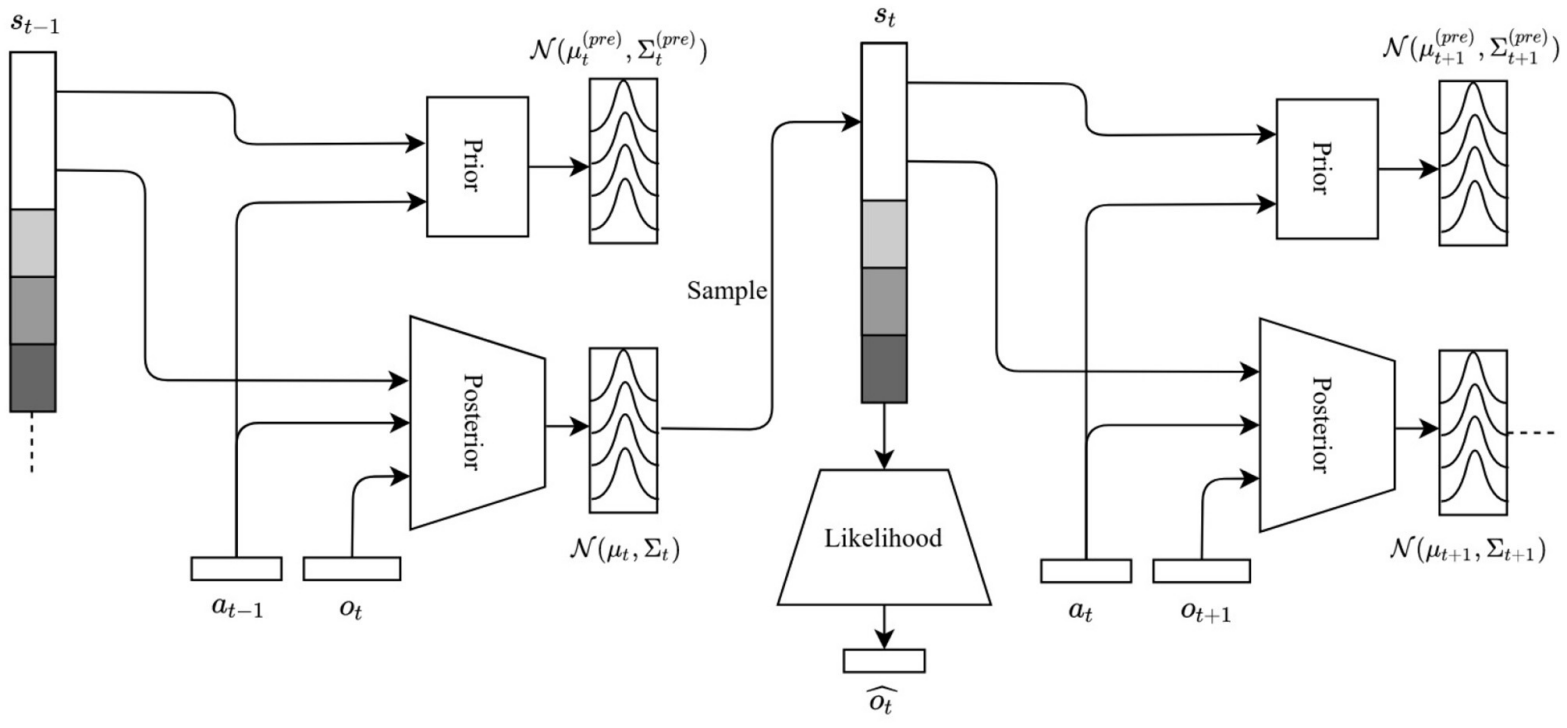
Information flow between neural networks in the deep active inference framework. The posterior network takes the current observation ***o***_*t*_, previous state ***s***_*t*−1_ and previous action ***a***_*t*−1_, and returns a multivariate normal posterior distribution from which the current state is sampled. The prior takes the previous state ***s***_*t*−1_ and previous action ***a***_*t*−1_, and returns a multivariate normal prior distribution. The likelihood takes the current state ***s***_*t*_ and returns a reconstructed observation $\hat{o_{t}}$. The process is recurrent and is repeated until the end of the sequence. States ***s***_*t*−1_ and ***s***_*t*_ are shaded to reflect the fact that the dimensionality of the latent space is a hyperparameter and may vary.

Deep neural networks are used to parameterize the approximate posterior *q*_***θ***_(***s***_*t*_|***s***_*t*−1_, ***a***_*t*−1_, ***o***_*t*_), prior *p*_***ϕ***_(***s***_*t*_|***s***_*t*−1_, ***a***_*t*−1_) and likelihood *p*_***ψ***_(***o***_*t*_|***s***_*t*_), where we have introduced the parameters ***θ***, ***ϕ*** and ***ψ***. In combination with Equation (2), minimization of free energy is realized using the loss function

(8) $$\begin{array}{r} L_{t}\left(\theta ,\phi ,\psi \right)={\text{D}}_{\text{KL}}\left(q_{\theta }\left(s_{t}|s_{t-1},a_{t-1},o_{t}\right)||p_{\phi }\left(s_{t}|s_{t-1},a_{t-1}\right)\right)\\ -\log p_{\psi }\left(o_{t}|s_{t}\right), \end{array}$$

which corresponds to the free energy of time step *t*. Here, the expectation from Equation (2) is carried out through minibatches as in Kingma and Welling (2014) and can therefore be dropped from Equation (8). In practice, the free energy is averaged out over all time steps, $L=\frac{1}{T}\overset{T}{\underset{t=1}{\sum }}L_{t}$, before adjusting the weights of the neural networks. Importantly, approximate posterior, prior, and likelihood distributions are modeled as multivariate normal distributions. The information flow in the network is visualized in Figure 1.

This architecture makes it possible to engage in planning as shown by Çatal et al. (2020). In brief, this requires generating imaginary rollouts using the learned prior model. Trajectories are then scored using expected free energy (as in Equation 4). In practice, planning occurs through iterative Monte Carlo sampling. For each policy π, *J* state trajectories of *K* time steps are sampled using the prior model. The expected free energy for each policy is, then, estimated using (Friston et al., 2021).

(9) $$\begin{array}{l} {\hat{G}}_{t}\left(\pi \right)=\overset{t+K}{\underset{\tau =t+1}{\sum }}{\text{D}}_{\text{KL}}\left(N\left(\mu_{{\hat{s}}_{\tau }},\sigma_{{\hat{s}}_{\tau }}\right)||P\left(s_{\tau }\right)\right)+\frac{1}{\rho }H\left(N\left(\mu_{{\hat{o}}_{\tau }},\sigma_{{\hat{o}}_{\tau }}\right)\right)\\ \;+\;\underset{\pi^{\prime }}{\sum }\sigma \left(-\gamma {\hat{G}}_{t+K}\left(\pi^{\prime }\right)\right){\hat{G}}_{t+K}\left(\pi^{\prime }\right), \end{array}$$

where $\mu_{{\hat{s}}_{\tau }}$ and $\sigma_{{\hat{s}}_{\tau }}^{2}$ are the batch mean and variance of the state samples ${\hat{s}}_{\tau }$, $\mu_{{\hat{o}}_{\tau }}$ and $\sigma_{{\hat{o}}_{\tau }}^{2}$ are the batch mean and variance of the corresponding observation samples ${\hat{o}}_{\tau }\sim p_{\psi }\left(\cdot |{\hat{s}}_{\tau }\right)$, σ is a softmax function, and ρ is a hyperparameter which allows tuning the precision of prior preferences over states in the future. The last term effectively implements a deep tree search over policies, where each path is effectively an accumulation of expected free energy over actions. This construction has the same format as a Bellman recursion, but, in this instance, it is recursion of expected free energy functionals of (Bayesian) beliefs about latent states and policies as opposed to value functions of states and policies.

Despite the parameterization of the posterior and prior, the issue remains that the size of the latent space must be specified. The size of the latent space is an important hyperparameter, since it can have critical consequences regarding model performance and resource usage. The free energy functional (Equation 2) emphasizes the importance of the accuracy-complexity trade-off in active inference. While the latent space dimensionality should be large enough to encode all the relevant information for the task at hand contained in the observations and actions, it should also be small enough to have low memory usage and promote generalization. This is especially important during planning, since this step is done in latent space and requires drawing multiple samples and evaluating multiple paths. The dimensionality is, therefore, a trade-off, and identifying the critical value is not trivial and often depends on the application at hand. Due to this, there is no one-size-fits-all value for the size of the latent space, and a sensible value must be found through different means. In practice, this often implies trial and error or a parameter sweep; both of which are suboptimal, since they require a lot of resources and unnecessary training loops. In the following sections, we describe two methods to resolve the issue.

### 3.3. Off-Line Sleep

A method that is able to prune the latent space is the sleep algorithm proposed by Wauthier et al. (2020). It allows the agent to initialize the model with a large number of latent space dimensions and, subsequently, sleep until the model can no longer reduce. The approach resembles biological sleep in the sense that it minimizes model complexity *post hoc* and in the absence of observations. Additionally, in the active inference framework, it can be compared with BMR, which provides an analytic approach to removing redundant model parameters or latent space dimensions. However, in the current implementation, the model must be retrained after each “reduction”. Importantly, this is an off-line method, as the reduction happens when the model is not training.

The off-line sleep method is based on singular value decomposition (SVD). Geometrically, singular values in SVD can be understood as the lengths of the semi-axes of the ellipsoid containing the data. The idea is that small singular values correspond to small semi-axes and, therefore, can be pruned, as these dimensions are not informative. Algorithm 1 sketches the workings of the method. We start by selecting a large initial number of latent space dimensions ν. This number will be reduced through the ensuing iteration. We train the model with ν latent space dimensions for *E* epochs using a data set consisting of sequences of actions and observations. After training, we generate *Nν* sequences of latent vectors from the model using the data set. From each sequence, we sample a vector to populate a square matrix ***A***_*i*_, i.e., each row is a latent space vector, in such a way that we obtain *N* square matrices. We perform SVD on each ***A***_*i*_ and find the number of singular values that are larger than the threshold value α. Then, we compute a new latent space dimensionality by averaging the number of remaining singular values over the *N* matrices and round off to the closest integer value. Next, we retrain the model with the new latent space dimensionality and repeat the process until the number of dimensions can no longer be reduced.

**Algorithm 1:**
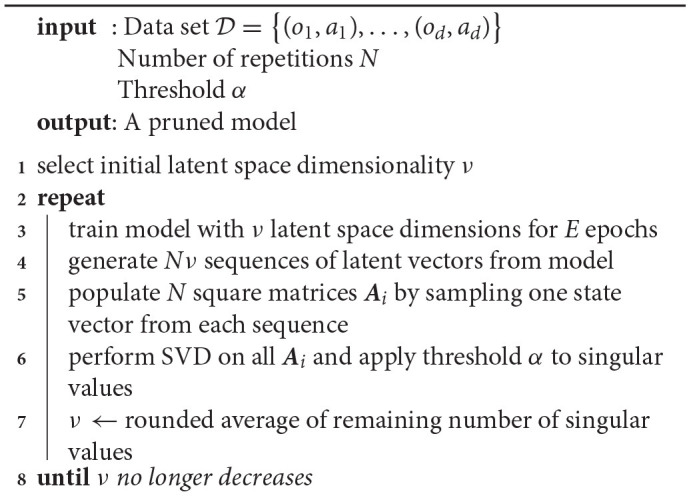
Off-line sleep on sequences of actions and observations.

Note that Algorithm 1 does not need to be executed in a single run. When training a model for a certain application, it is possible to pause after having trained the model a first time (line 3) and to continue whenever the application experiences downtime. This allows the model to be used from the start and be optimized in subsequent sleep runs.

Crucially, SVD does not allow one to know *which* dimensions can be pruned at each iteration. The method only indicates *how many* dimensions are informative. Geometrically, in general, the semi-axes of the earlier-mentioned ellipsoid are rotated with respect to the basis vectors of the latent space. As a result, singular values do not correspond to specific dimensions of the latent space and it is not possible to distinguish individual latent dimensions to prune. For this reason, retraining is a necessity. To alleviate this drawback, we now present an on-line method, which optimizes the number of latent dimensions as part of the training process.

### 3.4. On-Line Sleep

In response to the problem that the model must be retrained in off-line sleep, we present an on-line method for latent space pruning. In this method, weights are pruned on-the-fly during training. This type of model reduction is very similar to reflection, where the agent is allowed to reflect on its understanding of the observations during training. This results in “aha” moments: in a sense, moments where the agent realizes the world can be explained more simply. As we will show, this can be seen as sudden jumps in latent space dimensionality.

In brief, in order to reduce the number of states, we gate each state dimension with a gate parameter that follows a Bernoulli distribution. During optimization, we try to close as many of these gates as possible through *L*_0_ regularization. By co-optimizing the Bernoulli parameters in this manner, the model can learn how many states are required to solve a given task. At first glance, this poses two issues: first, the Bernoulli parameters make the gradient intractable and, second, without some way of limiting the reduction, the model will simply try to reduce the dimensionality to zero. The first issue can be resolved by making use of the Augment-REINFORCE-Merge (ARM) gradient estimator (Yin and Zhou, 2019). The second issue can be resolved through generalized ELBO with constraint optimization (GECO) (Rezende and Viola, 2018), as shown by De Boom et al. (2021). GECO introduces a Lagrange multiplier λ on the accuracy and a threshold hyperparameter τ on accuracy which controls λ. The accuracy term in the loss function (second term in Equation 8) will receive more weight during optimization as long as the desired level of accuracy has not been reached. Once the threshold has been reached, more weight will be given to the complexity term. In other words, the model must reach a certain level of accuracy before it can reduce its complexity.

One of the key features of working in the active inference framework is that data sets consist of sequences of actions and observations. This has a number of consequences for the implementation of the on-line sleep algorithm. For instance, whereas in a variational autoencoder (VAE) (Kingma and Welling, 2014) as demonstrated by De Boom et al. (2021), the gates are sampled with each forward pass through the posterior network, in deep active inference, the gates remain unchanged with each pass through the posterior as long as they relate to the same sequence. In other words, the gates are sampled once at the beginning of the sequence and are kept constant throughout the rest of the sequence.

Furthermore, without accounting for sequence length, loss values would increase with the length of the sequence. Therefore, it is necessary to average over sequence length in both the reconstruction error and KL divergence to compare sequences of different length. A direct result is that the interpretation of the threshold τ changes slightly, as it is now a threshold on the time-averaged reconstruction error.

Finally, note that the prior in a VAE is a simple multivariate standard normal, while in deep active inference, the prior is parameterized. Consequently, the posterior is no longer “pulled” toward a standard normal, and the KL divergence must be computed between the posterior and prior networks.

To summarize, the loss function at time step *t* becomes

(10) $$\begin{array}{l} L_{t}\left(\theta ,\phi ,\psi \right)=\frac{1}{\sum_{i}z_{i}}{\text{D}}_{\text{KL}}(q_{\theta }\left(s_{t}⊙z|s_{t-1}⊙z,a_{t-1},o_{t}\right)\\ \;||p_{\phi }\left(s_{t}⊙z|s_{t-1}⊙z,a_{t-1}\right))\\ \;-\lambda \left(-\log p_{\psi }\left(o_{t}|s_{t}⊙z\right)-\tau \right), \end{array}$$

where the gates ***z*** = **1**_[***u***<*S*(***ζ***)]_ with $u\sim \prod_{i=1}^{n}U_{\left[0,1\right]}$ are sampled once per sequence, *S*(ζ_*i*_) correspond to the Bernoulli parameters, λ is the Lagrange multiplier and τ is the accuracy threshold. Further, ⊙ denotes the Hadamard product and *S*(*x*) = (1 + exp(−*kx*))^−1^ is the sigmoid function with parameter *k*, where we set *k* = 7 as in Li and Ji (2020). We, once again, average over the sequence length and add the *L*_0_ regularization term, to obtain the loss function

(11) $$\begin{array}{l} L=\frac{1}{T}\overset{T}{\underset{t=1}{\sum }}L_{t}+\overset{n}{\underset{i=1}{\sum }}S\left(\zeta_{i}\right). \end{array}$$

Note that, similar to De Boom et al. (2021), we average the KL term over the number of open gates to ensure that having more states does not lead to a higher KL term, as this would lead to an additional influence on the dimensionality. Subsequently, the gradient for the gate parameters ζ_*i*_ becomes

(12) $$\begin{array}{l} \nabla_{\zeta^{ARM}}L=\frac{\lambda }{T}\overset{T}{\underset{t=1}{\sum }}\left(-\log p_{\psi }\left(o_{t}|s_{t}⊙\bar{z}\right)+\log p_{\psi }\left(o_{t}|s_{t}⊙z\right)\right)\\ \;\left(u-\frac{1}{2}\right)+\overset{n}{\underset{i=1}{\sum }}\nabla_{\zeta_{i}}S\left(\zeta_{i}\right), \end{array}$$

where $\bar{z}=1_{\left[u>S\left(\zeta \right)\right]}$.

Algorithm 2 sketches the workings of the on-line sleep method. As a result of gating and GECO, pruning of latent dimensions occurs within a single training loop and does not require multiple training loops. Initially, the model starts out with 95% of its gates open. It is then trained using Equation (11) *without regularization term* until the reconstruction error (accuracy) reaches the threshold value τ. Once the threshold has been reached, gates can be opened and closed. Practically, this means that, at this point, we start computing the gradient in Equation (12) and add the regularization term in Equation (11). Training thereupon continues until the model has trained a total of *E* epochs.

**Algorithm 2:**
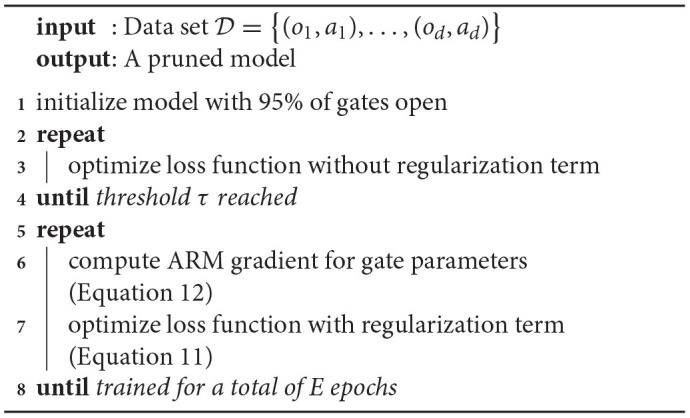
On-line sleep on sequences of actions and observations.

## 4. Results

### 4.1. Environments

**Figure 2:**
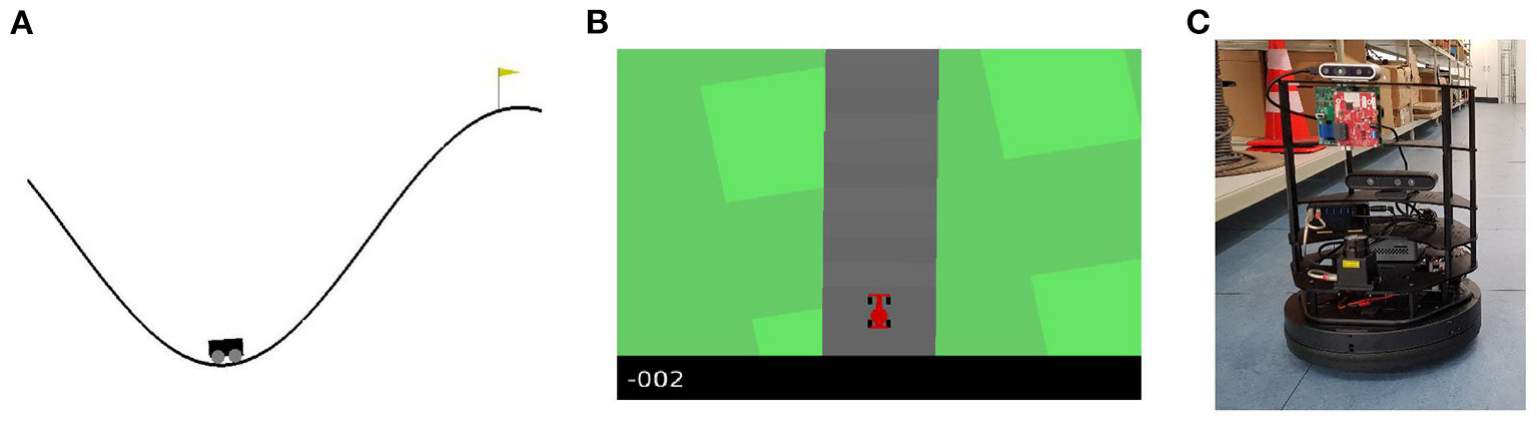
**(A)** In the mountain car environment a car can throttle left and right and needs to reach the hill top. **(B)** The carracer environment provides top-down pixel observations to race a car over a circuit. **(C)** The robot navigation scenario contains first person camera images and velocity control actions for a turtlebot driving around in a lab environment.

The methods presented in this article are evaluated using a number of different environments. The environments were selected to include a varying degree of difficulty. These include: the mountain car environment (Figure 2A), the car racing environment (Figure 2B) and a robot navigation environment in the lab on a turtlebot (Figure 2C). Here, the mountain car provides simple 1D observations, while the other environments provide pixel-based observations. Furthermore, while the mountain car and car racing environments are simulations, the robot navigation environment is real-life.

#### 4.1.1. Mountain Car

The mountain car environment is a classic, simple 1D environment. It consists of an underpowered car that starts in a valley and must drive up a steep mountain. Importantly, the car cannot drive up the mountain in one go and must build up momentum to reach the top of the mountain. The only actions it has, are accelerate left or right, or do nothing.

The original OpenAI gym environment (Brockman et al., 2016) returns observations with the position and velocity of the car. In our experiments, we employed a modified version in which only the position can be observed and noise is added to the observations, turning the problem into a POMDP. Due to the simplicity of the environment, we expect to only need 2 latent space dimensions to solve the problem, i.e., position and velocity. This allows for efficient evaluation of the proposed algorithms, since the expected dimensionality is already known.

The data set is generated before training using a random agent and consists of 100 sequences of length 200. When passing the data to the model during training, we randomly extract a subsequence of 100 consecutive steps out of each sequence. That way the model learns the dynamics of the environment. Similarly, the test data set was generated using a random agent and consists of 32 sequences of length 200.

#### 4.1.2. Car Racing

The car racing environment (Brockman et al., 2016) consists of a top-down view of a race car on a racetrack. The goal is for the race car to remain on the racetrack. In our implementation, the car can accelerate and turn right or left. The environment returns observations as 96 × 96 pixel images. In other words, observations are high-dimensional and the dynamics are more complicated than in the mountain car environment. In this case, the optimal number of latent dimensions cannot be known in advance.

In this case, the data set consists of 7 prerecorded sequences of differing lengths: 568, 723, 482, 521, 669, 647, and 676. When passing the data to the model during training, we randomly extract a subsequence of 15 consecutive steps out of each sequence. The test data set consists of a prerecorded sequence of length 635.

#### 4.1.3. Robot Navigation

Beside the previous environments, experiments were done on the mobile robot data set provided by Çatal et al. (2021). The data set consists of camera, lidar, and radar recordings of the industrial IoT lab at UGent. For our purposes, we only require the camera recordings which contain sequences of 240 × 320 pixel images. Again, this is a high-dimensional environment with complicated dynamics and the optimal number of latent dimensions is not known beforehand. Moreover, the data originate from the real world, albeit a controlled environment, and are not obtained from simulation.

### 4.2. Mountain Car

By virtue of the simplicity of the mountain car environment, we use this environment to inspect whether our methods are able to converge to the optimal dimensionality, i.e., 2 dimensions. Effective reduction, for both off-line and on-line sleep, depends on the chosen threshold, α and τ, respectively.

Artificial neural networks are instantiated as in Çatal et al. (2020). That is, prior, posterior and likelihood networks consist of fully connected neural networks with two hidden layers which each contain 20 neurons. Importantly, the size of the output layer of the prior and posterior networks equals the size of the latent space, and is therefore selected by the model reduction algorithm. This also applies to the input layer of the likelihood network. Baseline and off-line sleep runs are trained with a batch size of 64 sequences of length 100 using the loss function in Equation (8) with an additional factor 2 on the complexity term and an additional regularization term with weight 0.001 comprised of the KL divergence between the posterior and a standard normal distribution. On-line sleep runs are trained with a batch size of 32 sequences of length 100 using the loss function in Equation (11). All minimization occurs through the Adam optimizer with learning rate 0.001. Additional details are described in Çatal et al. (2020).

**Figure 3:**
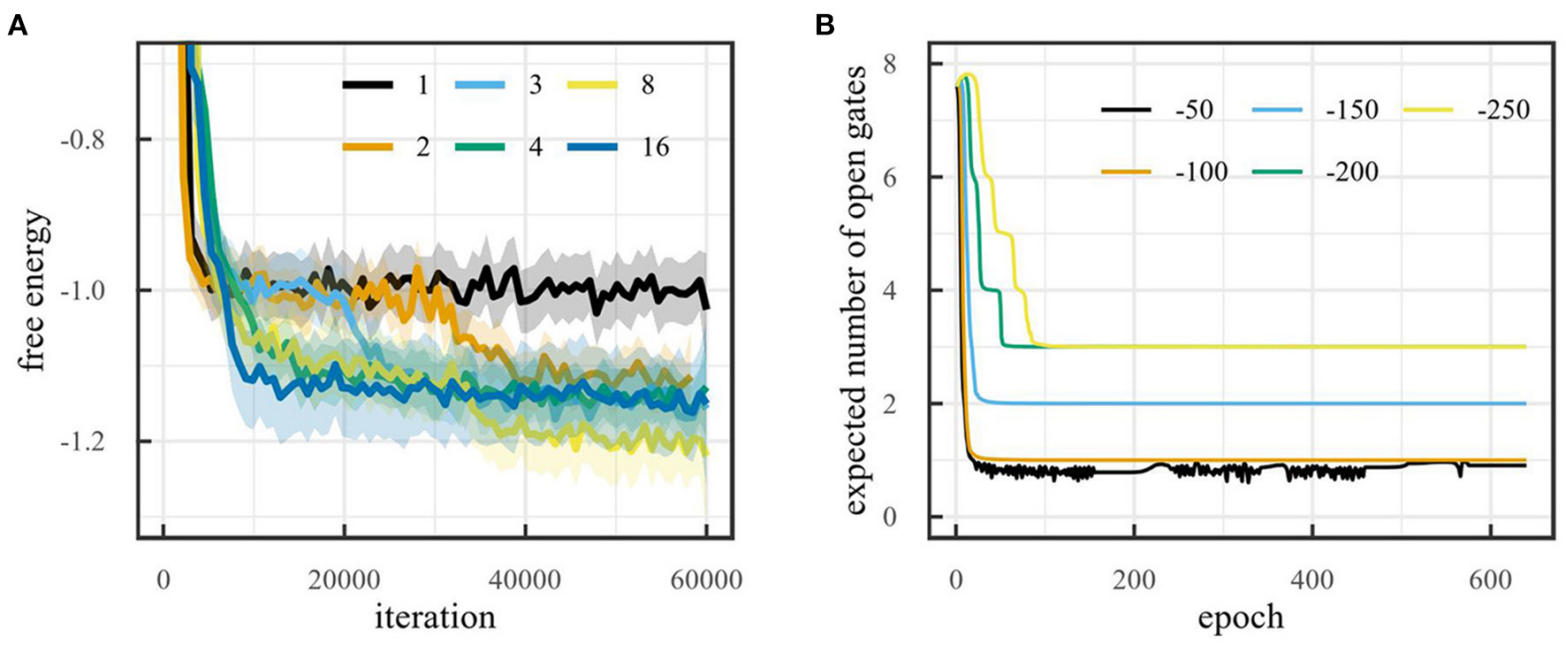
**(A)** Free energy during training on the mountain car environment for different latent space sizes (LOESS smoothed, span 0.02). **(B)** Number of dimensions obtained with on-line sleep on the mountain car environment for different thresholds with initial number of dimensions 32.

As mentioned in Section 4.1.1, the environment can be solved using two latent dimensions i.e., position and velocity. As a result, we expect to find a lower free energy when the model has more than one latent dimensions, since a latent space with only one dimension can only encode the position, but never the velocity, while a latent space with more than one dimension can encode position, velocity and (possibly) noise on the observations. Figure 3A shows the free energy during training for the mountain car environment for different latent space dimensionalities. The figure shows that, after 60,000 training iterations, the free energy for models with one latent dimension is indeed significantly higher than for models with more than one dimension. Note that the free energy for models with more than one latent dimension does not decrease significantly when increasing the number of dimensions. Small differences in free energy are likely due to differences in how noise is encoded. If the noise characteristics are better encoded, this can lead to a slightly lower free energy.

**Figure 4:**
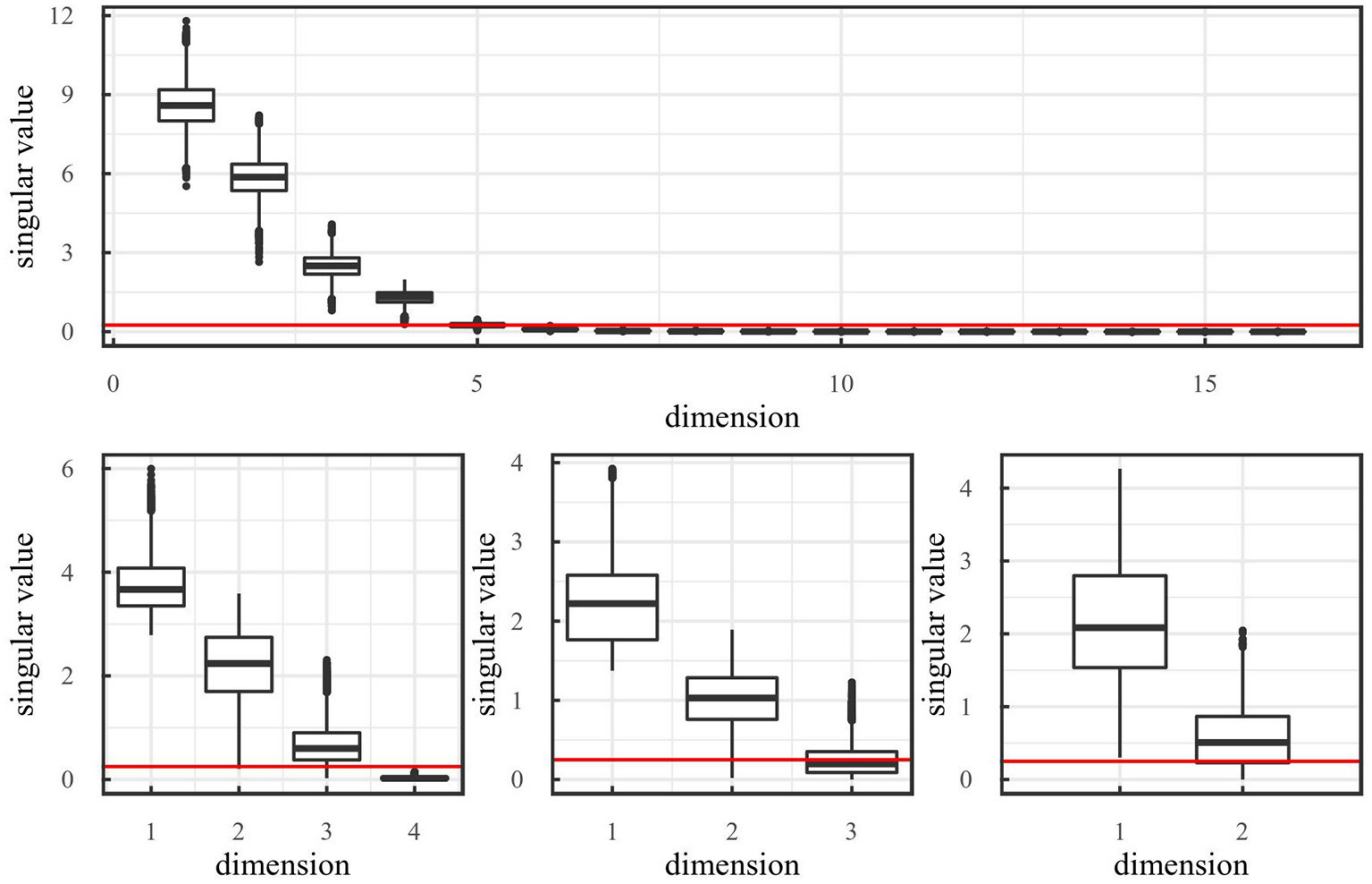
Boxplots of singular values after off-line sleep on the mountain car environment for different dimensionality. The red line indicates a threshold α = 0.25. These figures show that sleeping with the chosen threshold would result in the reduction: 16 → 4 → 3 → 2.

Figure 4 shows singular values from off-line sleep after training on the mountain car environment. The figure also shows that performing off-line sleep with threshold α = 0.25 results in a reduction from 16 dimensions to 2 dimensions through 16 → 4 → 3 → 2.

Figure 3B shows the number of dimensions obtained for the mountain car environment for different values of the threshold τ when the initial number of dimensions is 8. It also shows how final dimensionality depends on the threshold. This makes sense, when the threshold is set too low, the model will attempt to obtain a very high accuracy by modeling noise, increasing the dimensionality. When the threshold is set too high, not enough information will be encoded in the latent space and the dimensionality will continue to reduce. Note that for τ = −50, the algorithm continues to attempt to reduce the dimensionality to 0, but since this would lead to a very low accuracy, the dimensionality shoots back up to 1.

### 4.3. Car Racing

Since the car racing environment provides high-dimensional observations, it is a useful environment to assess the model's performance. We assess the evolution of the model's performance both during and after training.

Again, neural networks are instantiated as in Çatal et al. (2020). The posterior network consists of a fully connected neural network, where feature vectors are extracted from observations by convolutional layers and concatenated to action and state vectors. Consequently, the likelihood network consists of a deconvolutional neural network. Contrary to the architecture for the car racing environment in Çatal et al. (2020), the prior consists of an LSTM with 128 features to allow for more temporal depth in the prior transition model. As in the case with the mountain car, the size of the output layer of the prior and posterior networks and the size of the input layer of the likelihood network equal the size of the latent space. More details on the network and architecture can be found in Supplementary Material.

**Figure 5:**
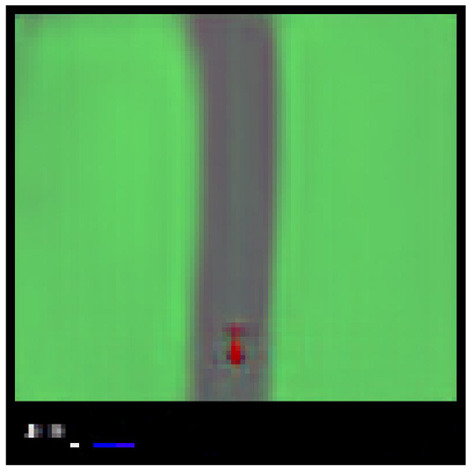
Reconstruction of preferred state used for car racing environment evaluations.

To assess model performance, the preferred state is defined by taking an observation in which the car is located in the middle of the track and translating it to state space (see Figure 5). In practice, the environment is always initialized with the car in the middle of the road, therefore, the first frame can be taken as preferred observation. Performance is then evaluated through an active inference agent with this preferred state and ρ = 0.0001.

Additional details on neural network architecture and model performance assessment are described in Çatal et al. (2020).

#### 4.3.1. Off-Line Sleep

**Figure 6:**
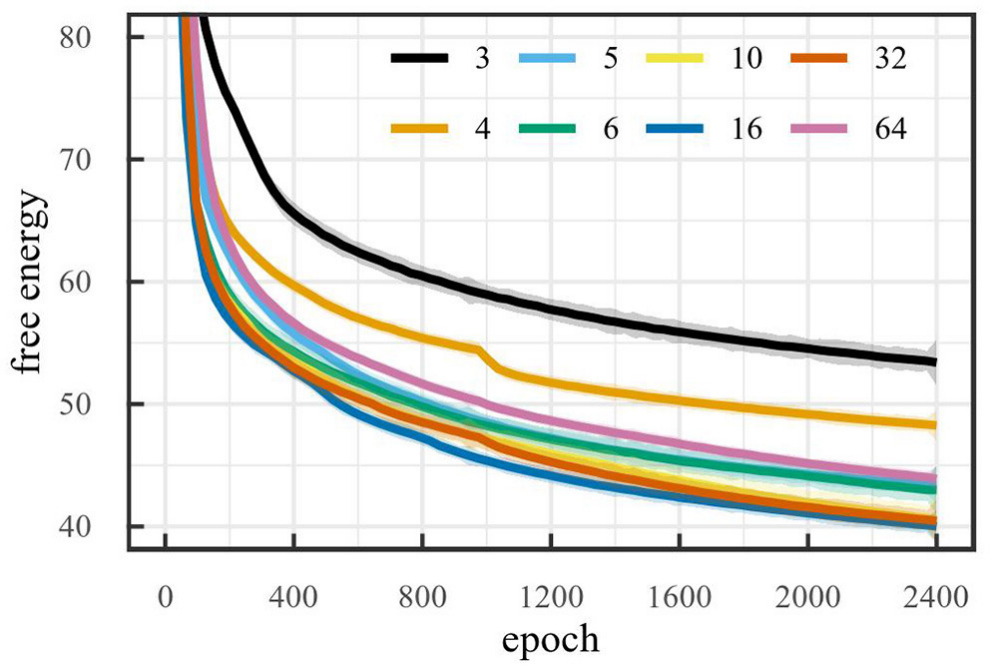
Free energy during training on the car racing environment for baselines runs with different latent space sizes (LOESS smoothed, span 0.02).

Figure 6 shows the free energy during training for the car racing environment. To make sure the latent space retains all information after pruning, it is important that the free energy remain minimal. Note that the free energy does not decrease for latent space dimensionality larger than 5. That is, adding dimensions, when the dimensionality is larger than or equal to 5, does not reduce the free energy. Inversely, removing dimensions, when the dimensionality is less than or equal to 5, increases the free energy. This suggests that the optimal latent space dimensionality is 5, since this is the smallest value for which the free energy remains minimal. As a result, this is the critical value which the algorithm should attempt to achieve.

**Figure 7:**
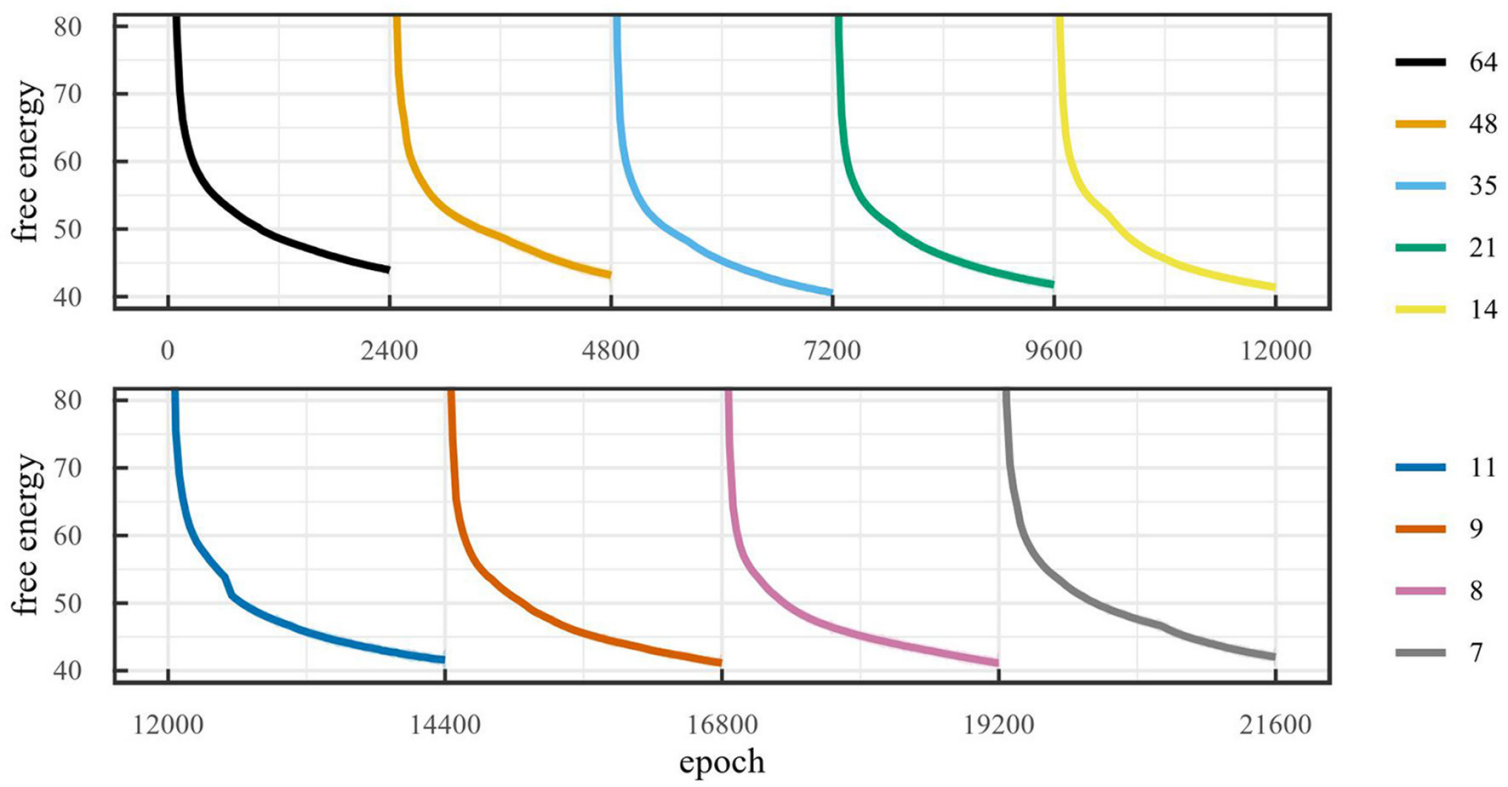
Free energy during training over an off-line sleep process with α = 0.1 for the car racing environment (LOESS smoothed, span 0.02). This run required 9 cycles of off-line sleep and converged to 7 dimensions, where the model was trained for 2,400 epochs each cycle.

Figure 7 demonstrates the workings of the off-line sleep algorithm. After training for 2,400 epochs, the model performs SVD in an attempt to reduce the dimensionality. If the dimensionality can be reduced, the excessive dimensions are pruned and the model retrains with a reduced number of dimensions, otherwise training stops.

#### 4.3.2. On-Line Sleep

Since the regularization term consists of the sum of the Bernoulli parameters of each gate, this term also indicates how many gates are expected to remain open. Therefore, we use this number to indicate the latent space dimensionality in the on-line sleep case.

**Figure 8:**
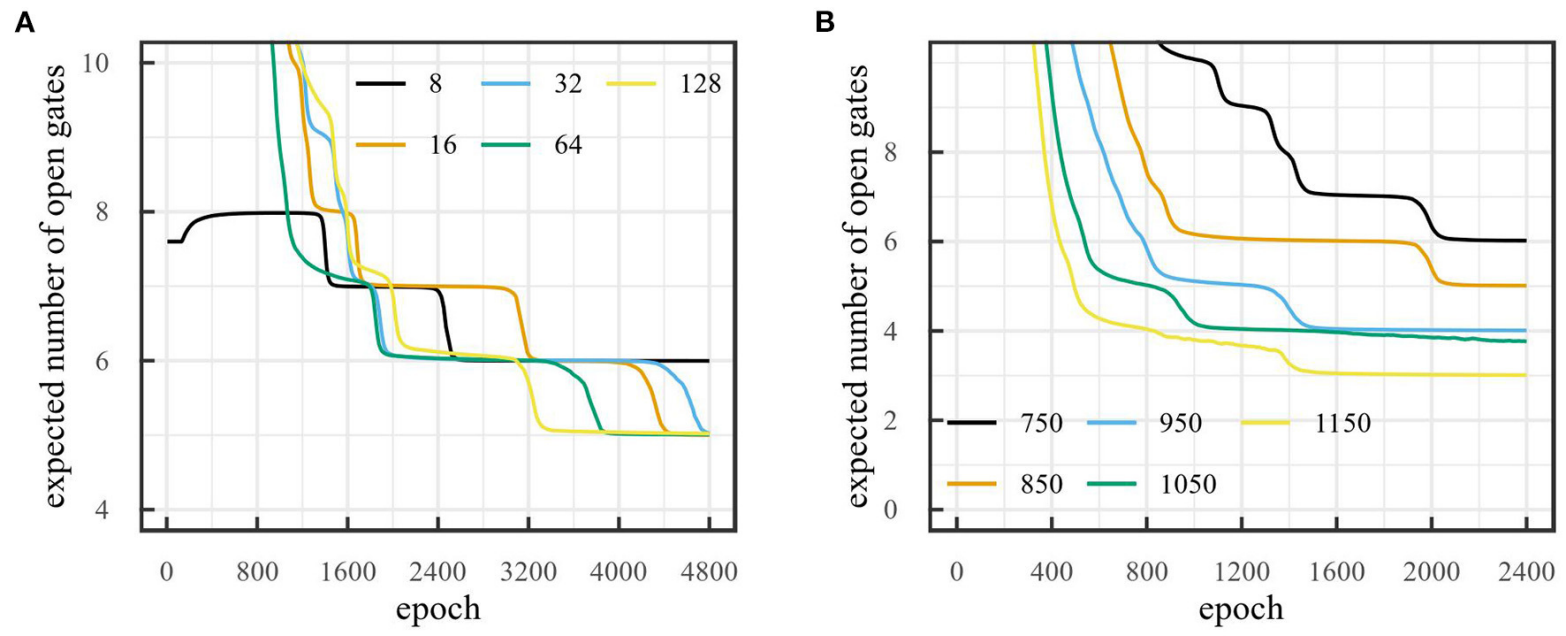
Number of dimensions obtained with on-line sleep on the car racing environment **(A)** for different initial number of states with threshold τ = 800 over 4,800 epochs and **(B)** for different thresholds with initial number of dimensions 32 over 2,400 epochs.

Since initialization is important in the convergence of neural networks, it is important to assess the impact of different initial values on the final dimensionality. Two hyperparameters are crucial here: initial dimensionality and threshold value τ. Firstly, we investigate the effect of initial dimensionality on final dimensionality when τ is fixed. Figure 8A shows the number of dimensions obtained for the car racing environment for different initial dimensionalities when the threshold is fixed to 800. The figure shows that after 4,800 epochs the number of gates that remain open is 5 for most runs when τ = 800. This suggests that final dimensionality is largely independent of initial dimensionality. The chosen threshold was based on the reconstruction error obtained after training the model without pruning. For this, we determined at which point the reconstructions became good for the human eye. It is likely that the reconstructions are good enough to evaluate policies before they become good for the human eye. However, the chosen threshold works well for our intents and purposes.

**Figure 9:**
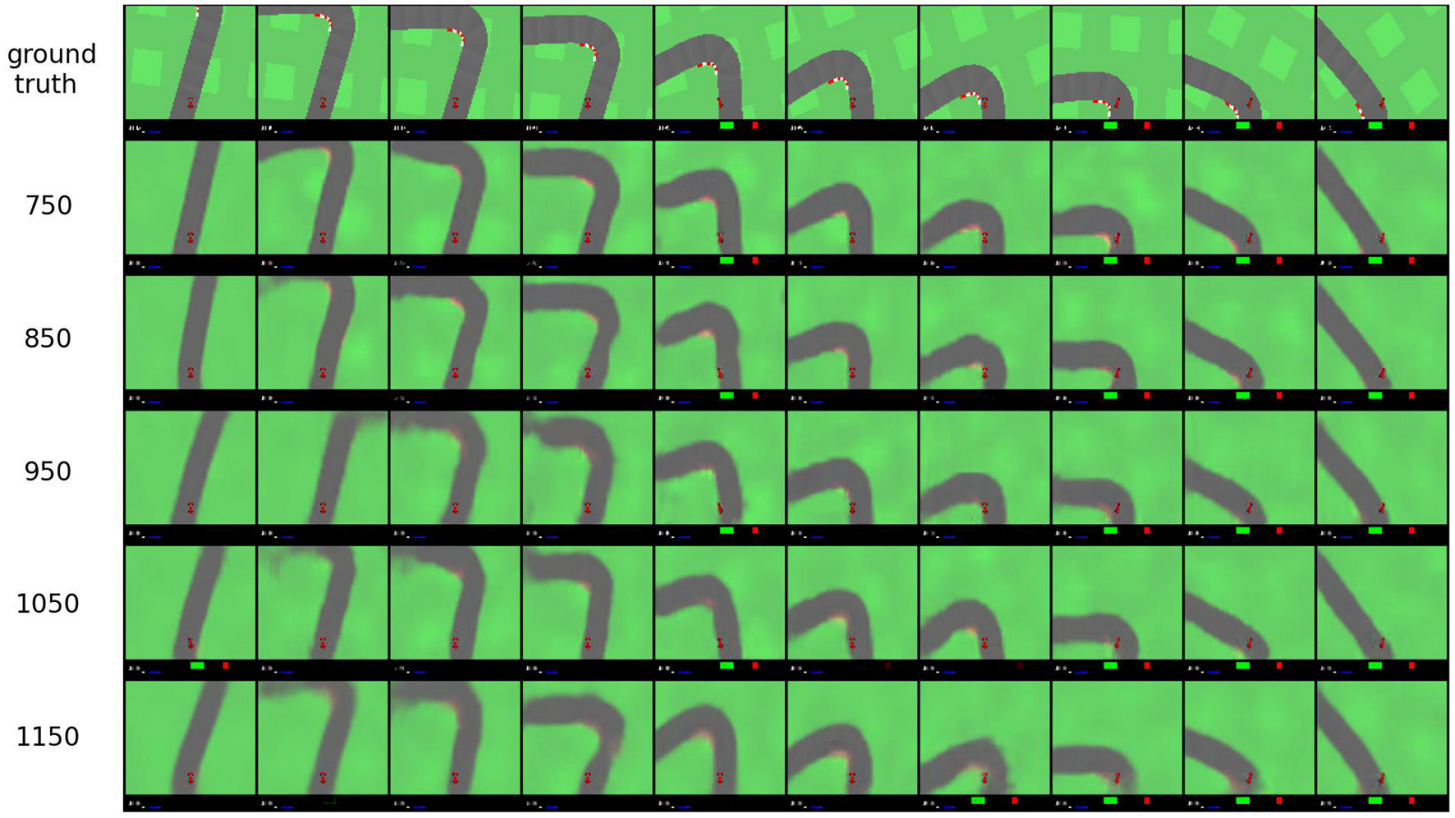
Reconstructed observations from the car racing environment after on-line sleep (32 initial dimensions, 2,400 epochs) with varying threshold τ (from **top** to **bottom**: ground truth, 750, 850, 950, 1,050, 1,150).

Secondly, we investigate the effect of the threshold value τ on final dimensionality. Figure 8B shows the number of dimensions obtained for the car racing environment for different values of the threshold τ when the initial number of dimensions is 32. This figure shows the dependency of the number of dimensions on the reconstruction threshold. A lower threshold leads to a higher dimensionality and vice-versa. Furthermore, Figure 9 illustrates how the reconstructed observations change depending on threshold τ. A higher threshold tends to lead to a slightly blurrier image and less accurate image features.

**Figure 10:**
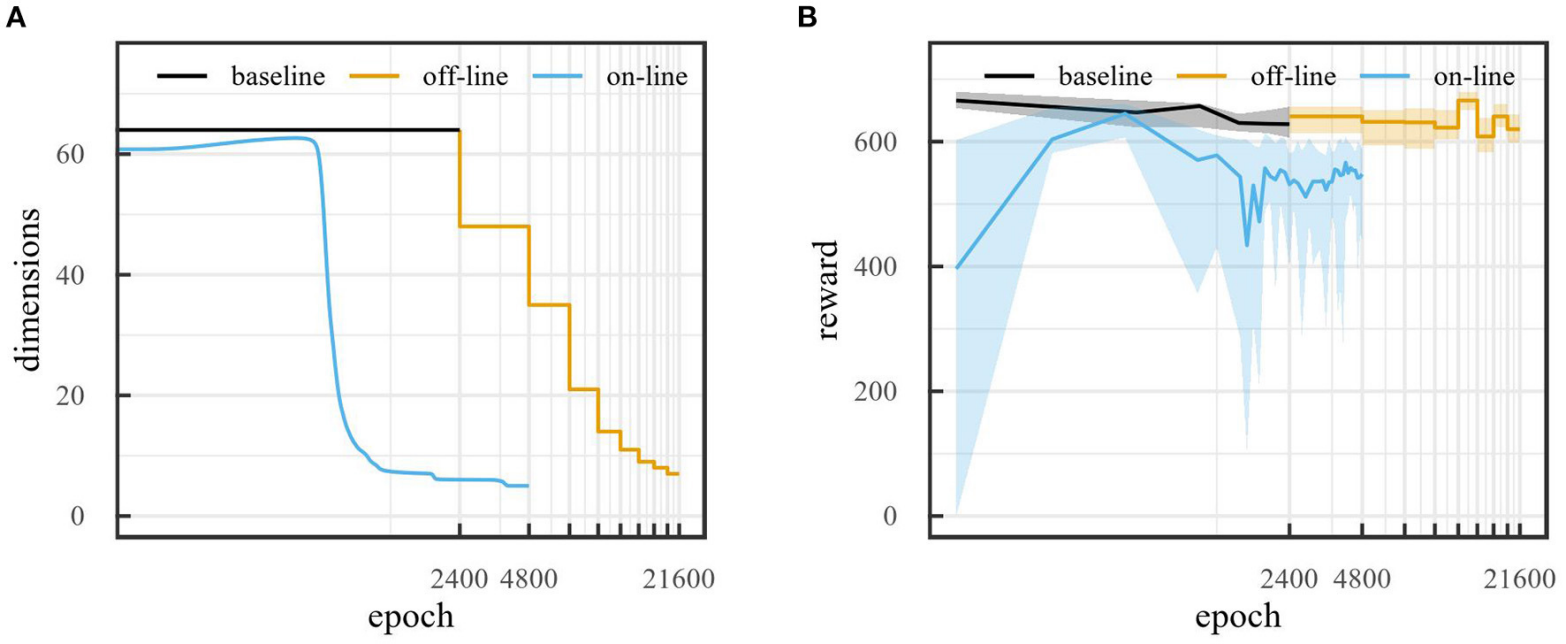
Evolution of **(A)** number of states and **(B)** median reward obtained (over 20 rollouts) over number of epochs for baseline, off-line sleep (α = 0.1), and on-line sleep (τ = 800) runs with 64 states initial latent space dimensions. Error bars show 25 and 75% quantiles. For off-line sleep, each step of the reward curve shows the median reward obtained at the end of the training run for the corresponding dimensionality, i.e., after training for 2,400 epochs at the corresponding dimensionality.

To evaluate how the model performs, we used the reward obtained from the car racing environment. This reward increases with 1, 000/*R* with every new track tile visited, where *R* is the total number of track tiles, and decreases by 0.1 per frame. If the car exits the playing field, it loses 1, 000 reward per frame instead. If the reward drops below 0, we consider the rollout as failed and set the reward to 0. We used a rollout length of 300 time steps. Figure 10A compares the evolution of the number of latent space states during baseline runs, off-line sleep with α = 0.1 and on-line sleep with τ = 800 for 64 initial latent space dimensions. Both off-line and on-line sleep are able to effectively reduce the dimensionality of the latent space. However, on-line sleep manages to reduce the latent space to 5 dimensions over 4,800 epochs, while off-line sleep requires 21,600 epochs to reduce to 7 dimensions. Furthermore, Figure 10B compares the evolution of the reward during the same runs. Importantly, for off-line sleep, each step of the reward curve shows the average reward obtained at the end of the training run for the corresponding dimensionality, i.e., after training for 2,400 epochs at the corresponding dimensionality. The performance of the off-line sleep run remains in range of the baseline run. This makes sense, as off-line sleep can simply be seen as a concatenation of baseline runs with decreasing dimensionalities. In comparison, the performance of the on-line sleep run diminishes slightly during training. The lower performance can be attributed to the fact that the model needs to adapt to less dimensions each time the dimensionality reduces.

### 4.4. Robot Navigation

To score performance in this real-world application, we compare the reconstructed observations from full (baseline) and reduced models as a proxy for model evidence (c.f., classification accuracy based upon posterior predictive densities).

The architecture is similar to the one used for the car racing environment. Details on the layers can be found in the Supplementary Material. Again, the size of the output layer of the prior and posterior networks and the size of the input layer of the likelihood network equal the size of the latent space. Models are trained with a batch size of 8 sequences of length 10. Loss function minimization occurs through the Adam optimizer with learning rate 0.0001.

**Figure 11:**
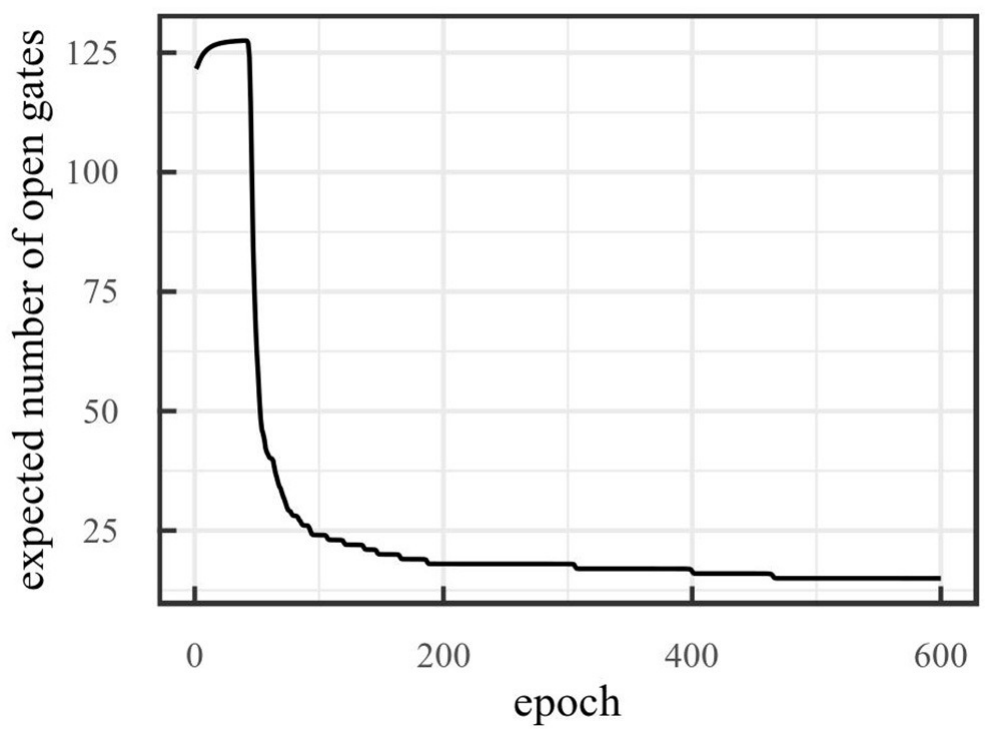
Number of dimensions obtained with on-line sleep on the robot navigation environment with threshold τ = 12,000. After 600 epochs, reduction stopped at 15 dimensions.

Figure 11 shows the number of dimensions obtained with on-line sleep on the robot navigation environment with τ = 12,000 and an initial dimensionality of 128. Over 600 epochs, the number of dimensions reduces to 15. Again, the threshold was based on the reconstruction error obtained after training the model without pruning. We determined at which point the reconstructions became good for the human eye.

**Figure 12:**
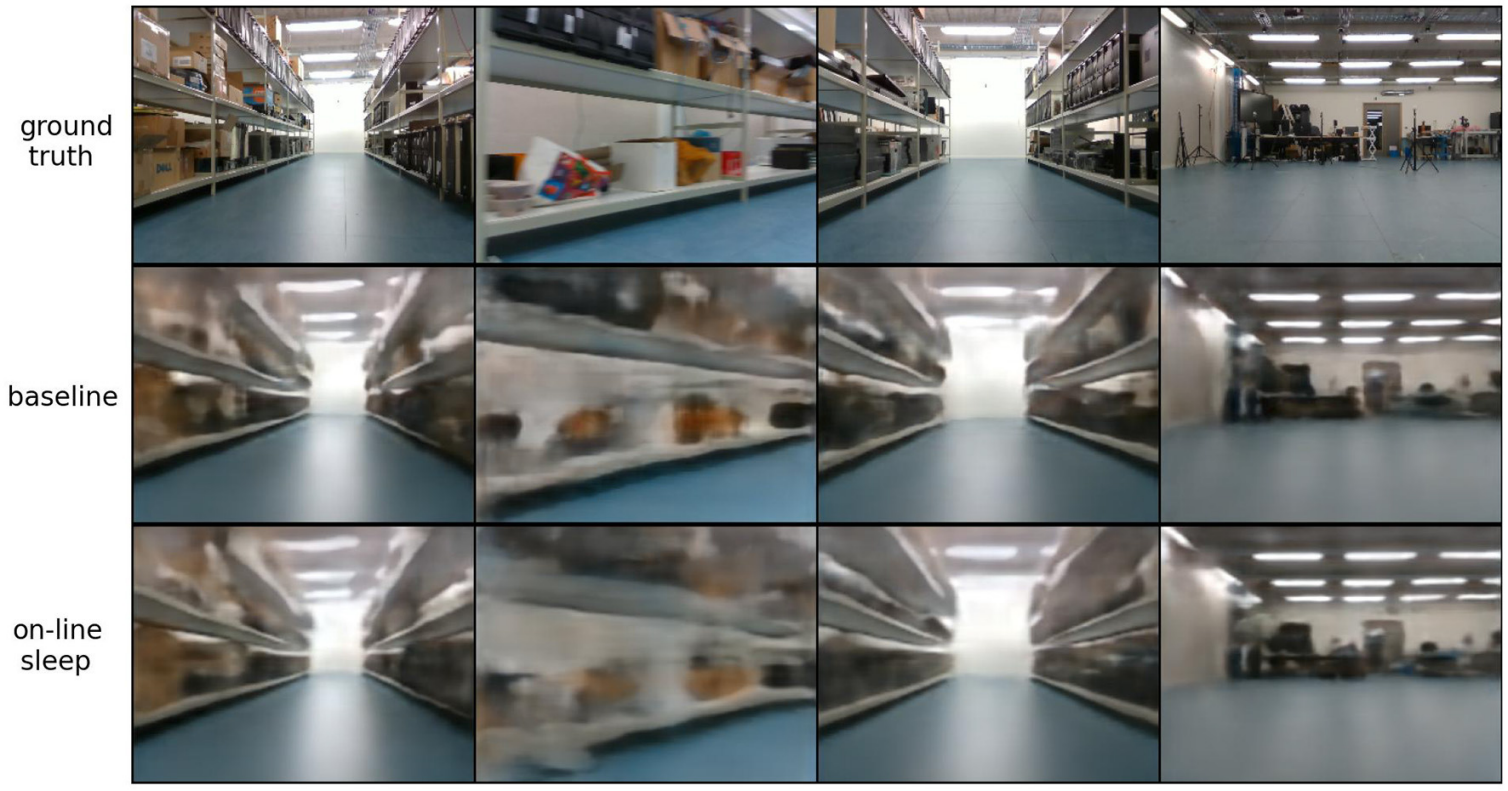
Reconstructed observations on the robot navigation environment. The top row contains ground truth observations, the second row contains reconstructions for a model trained with 128 latent space dimensions (achieving a reconstruction error of 9,460), and the third row shows reconstructions for a model trained with on-line sleep with τ = 12,000 (see Figure 11).

Reconstructed observations are shown in Figure 12. Here, the top row shows ground truth observations, the second row shows reconstructions for a model trained with 128 latent space dimensions (which reaches a reconstruction error of 9,460), and the third row shows reconstructions for the model trained with on-line sleep (at τ = 12,000) displayed in Figure 11. No significant differences are visible, suggesting that while on-line sleep is able to reduce the number of latent space dimensions, it does not significantly reduce the quality of the reconstructions. In other words, on-line sleep enables us to obtain a quality of reconstructions similar to baseline using fewer latent space dimensions.

## 5. Discussion

The results show that both methods are able to effectively prune dimensions in the latent space of a deep active inference model. However, there is an important trade-off. While the off-line sleep method requires multiple training runs to converge, on-line sleep only requires a single training run. On the other hand, while the performance can reduce slightly during on-line sleep, each off-line sleep cycle trains for a specific latent space dimensionality and therefore maintains performance.

Performance evaluations show that there is indeed a slight decline in performance when using on-line sleep compared to baseline training runs. This decline can be attributed to the small number of epochs that the model trains for a certain dimensionality. While baseline runs have 2,400 epochs to optimize for a given dimensionality, on-line sleep runs continually reduce dimensionality and, therefore, must continually adjust to the dimensionality. This could potentially be resolved by, once again, training without regularization and without updating the gate parameters after the model has finished training.

Results from on-line sleep show that the final dimensionality is robust with respect to initial dimensionality settings of the algorithm. Any sufficiently large initial state space dimensionality will lead to the same final dimensionality, given that the model has trained long enough. This result makes sense, since the initial dimensionality does not determine which or how much information should be encoded in the latent space. Instead, it simply provides the model with a larger initial latent space in which to encode information. Excess dimensions are then pruned by the algorithm. Conversely, final dimensionality does depend on the chosen threshold value. Indeed, the reconstruction threshold determines how much detail from the observations should be retained. A low threshold ensures more detail is retained, which in turn leads to more dimensions, and vice-versa. Based on these results, a possible course of action would be to first train a model without sleep and a sufficiently large dimensionality in order to find a good reconstruction threshold, and finally, train a model with on-line sleep using the previously obtained threshold and a sufficiently large dimensionality.

Moreover, in this work, the measure for adapting the Lagrange multiplier is the level of accuracy or reconstruction error. It may be possible to use other measures. For example, in the case of the car racing environment, a more sensible approach to threshold selection could be to adapt the threshold based on the performance of the model, in such a way that the model will only reduce if the performance can be maintained. That is, as long as the performance does not reach the same level as before reduction, it should not reduce further. This is a research route that will be explored in future work.

The number of epochs is also important for the final dimensionality, because the dimensionality depends on the number of epochs. One could inquire until what point models should be trained. In the end, the purpose of on-line sleep is to be able to learn a task and prune dimensions simultaneously so as not to require spending (much) more time finding an optimal latent space dimensionality through parameters sweeps or off-line sleep. Observing that final dimensionality is largely independent of initial dimensionality, it may be good practice to spend around the same number of epochs as a baseline run would require to converge.

Additionally, it is important to discuss the role that the data (and the environment) play. In any type of learning, the data are an integral part of the final result. For example, in deep learning applications, overrepresentation of a certain aspect of the data can lead to biases in the model. In our case, the data have an effect on the final dimensionality. For the car racing example, final dimensionality may depend on the complexity of the observations given to the model. Observations that contain more complex tracks could lead to more dimensions being necessary.

Off-line sleep can be compared to more traditional dimensionality reduction techniques, e.g., principal component analysis (PCA), non-negative matrix factorization (NMF), etc., in the sense that it reduces the state space *post hoc* by evaluating linear combinations and correlations between dimensions. Unfortunately, this method can only be applied after the model has been trained, which can cause the reduction process to take a considerable amount of time, since the model must be retrained. On-line sleep, instead, is able to perform model reduction while the model is training. This removes the need to retrain.

Reduction methods for MDPs mentioned in the literature are typically designed to maintain the structure of the state space after reduction. However, since our methods learn the state space through neural networks, it is not necessary to maintain this structure. Indeed, in the case of off-line sleep, a simple SVD suffices, since the model must retrain after reduction. In fact, any method that removes dimensions from the state space, i.e., prunes connections in the neural network, requires that the network be retrained (or further trained), which defeats the purpose of maintaining structure. In addition, since the structure of the state space changes during training, most methods cannot be used during training and must be applied *post hoc*. In the case of on-line sleep, the model is trained during reduction, which means it adjusts itself to the number of remaining dimensions on-the-fly.

Crucially, the main increase in difficulty in the environments throughout this work consists of the complexity of the observations. The complexity increased from 1-dimensional observations for the mountain car to high-dimensional rendered images for car racing, and high-dimensional real world images for the robot. In addition to complexity in observation space, one could consider increasingly complex tasks that need to be executed, i.e., increasing action spaces. In this case, an optimal dimensionality and structure of the state (and action) space could have an effect on the agent performance, for example in the context of hierarchical reinforcement learning (Asadi and Huber, 2004). However, this comparison will be further explored in future work.

Finally, in this work, we investigate the latent space size in relation to active inference. However, deep active inference introduces neural networks, where the size of each layer must also be specified. In the future, we will work on applying the gating mechanism to layers in the neural network.

Although not pursued in this work, the use of Bayesian model reduction also finesses the problem of sharp minima in neural networks by automatically minimizing model complexity. This is a subtle issue in the sense that most approaches to eluding local minima involve adding extra parameters to destroy fixed points on (e.g., variational free) energy landscapes. However, having escaped local minima, it is then necessary to prune the network to ensure generalization.

### 5.1. Bayesian Model Reduction

Bayesian model reduction is a relatively new procedure that can be regarded as a special case of Bayesian model selection; namely, selecting the model with the greatest marginal likelihood or model evidence. The particular benefit of BMR is that the evidence for a reduced model can be evaluated analytically, given the evidence and posteriors of a parent or full model. This means that models can be scored in terms of their evidence quickly and efficiently, without any need for retraining, having learned the full model. This efficient form of model selection rests on casting reduced models as models in which certain parameters (i.e., connections) are removed using precise shrinkage priors. It can be regarded as a generalization of the Savage-Dickie ratio for automatic model selection (c.f., automatic relevance detection). Commonly used equations for BMR can be found in Friston et al. (2019) for a variety of distributions over model parameters (ranging from Gaussian densities to Dirichlet distributions).

To leverage the efficiency of BMR, it is necessary to have a posterior over the model parameters that need pruning. This presents a slight problem for deep active inference, because we have replaced the parameters of a generative model with neural networks with learnable weights that are treated as point estimators, with no uncertainty. In vanilla treatments of active inference, the approximate posterior covers latent states, policies and parameters. Conversely, in deep active inference we only have posteriors over the latent states and policies. To finesse this problem, we have equipped the parameters with switching or gating variables that play the role of sufficient statistics of a simple posterior over model parameters (i.e., the connection weights).

We can now conjecture that the reduced variational free energy of the implicit posterior over model parameters, under different reduced models, can be approximated with the GECO; namely, a generalized ELBO with constraint optimization. If this conjecture is true, it suggests that the online sleep procedure described above is, the Bayes optimal way to prune networks. As with all BMR procedures, there remains the delicate issue of how much data to acquire (i.e., the duration of the training periods), before running BMR. This is usually dictated by the trade-off mentioned above, in terms of the speed of optimization, relative to the amount of time it takes. An upper limit on the amount of training or data is clearly provided by the times over which the model (i.e., generative process) does not change. Heuristically, a lower limit depends on how much evidence is required to commit to a simpler model.

## 6. Conclusion

In this article, we have examined and compared two methods that are able to reduce the latent space in deep active inference models: an off-line method that reduces a trained model *post hoc* and reapplied multiple times, and an on-line method which applies a gate to each dimension in the latent space and can prune dimensions on-the-fly during training.

Experiments on the mountain car environment showed that both methods were able to achieve the optimal number of dimensions depending on the chosen threshold. Results from the car racing environment showed that the off-line method was able to retain performance, but used approximately five times more epochs to converge, while the on-line method showed slightly lower performance. Finally, the robot navigation environment showed that even in real-world settings on-line sleep was able to reduce dimensionality.

### 6.1. Future Work

Aside from the ideas already discussed in section 5, we would like to explore one more point of interest: model expansion. This work showed that it is possible to reduce latent space dimensionality. A question that arises is what happens when the agent obtains entirely new observations. One would expect the state space to expand, since it must accommodate for new information. Future work will call attention to this topic.

## Data Availability Statement

The raw data supporting the conclusions of this article will be made available by the authors, without undue reservation.

## Author Contributions

SW and CD worked out the mathematical basis for the experiments. SW, CD, TV, and BD conceived the experiments. SW performed the experiments. All authors supervised the experiments. All authors contributed to the article and approved the submission.

## Funding

This research received funding from the Flemish Government under the Onderzoeksprogramma Artificiële Intelligentie (AI) Vlaanderen program. OÇ was funded by a Ph.D. grant of the Flanders Research Foundation (FWO).

## Conflict of Interest

The authors declare that the research was conducted in the absence of any commercial or financial relationships that could be construed as a potential conflict of interest.

## Publisher's Note

All claims expressed in this article are solely those of the authors and do not necessarily represent those of their affiliated organizations, or those of the publisher, the editors and the reviewers. Any product that may be evaluated in this article, or claim that may be made by its manufacturer, is not guaranteed or endorsed by the publisher.
