# Supplementary material for: Model Reduction Through Progressive Latent Space Pruning in Deep Active Inference
Source: Front Neurorobot. 2022 Mar 11;16:795846. doi: 10.3389/fnbot.2022.795846 (PMC8961807; doi:10.3389/fnbot.2022.795846)
Supplement: Supplementary file 1 [file Data_Sheet_1.PDF]

Supplementary Material

1 SUPPLEMENTARY TABLES AND FIGURES

1.1 Neural architectures

Table S1. Neural architectures for the car racing (CR) and robot navigation (RN) environments.

Network	Layer	CR neurons/filters	RN neurons/filters
Posterior	Convolutional	8	8
			16
		16	32
			32
		32	64
			64
		64	128
			128
		128	256
	Concat		
	Linear	128	128
	Linear	$2 \times s$	$2 \times s$
Likelihood	Linear	128	128
	Linear	$128 \times 3 \times 3$	$256 \times 4 \times 5$
	Convolutional	128	256
			128
		64	128
			64
		32	64
			32
		16	32
			16
		8	8
			8
Prior	LSTM cell	128	128
	Linear	$2 \times s$	$2 \times s$

Convolutional layers have stride 1, padding 1 and filter size 3×3. Upsampling in the likelihood occurs through nearest neighbor interpolation after each convolutional layer. The "Concat" step creates a vector by concatenating the observation feature vector obtained from the convolutional pipeline with an action vector and latent space vector. All Linear and Convolutional layers have leaky ReLU activation functions. Concat has no activation. Variance output (denoted by $2\times$) uses a softplus activation.
